# Supplementary material for: Integrative analysis of blood biomarkers and clinical variables improves early detection of aggressive prostate cancer
Source: Sci Rep. 2025 Apr 23;15:14071. doi: 10.1038/s41598-025-98980-3 (PMC12018954; doi:10.1038/s41598-025-98980-3)
Supplement: Supplementary file 1 — Supplementary Material 1 [file 41598_2025_98980_MOESM1_ESM.pdf]

Supplementary table 1 – Parameters, full p-values and patients' count

Parameter	p-value	p-value corrected	Type	Patients tested
Glucose	0.25750	0.51499	Laboratory measurement	334
Alkaline Phosphatase	0.47754	0.70374	Laboratory measurement	318
Triglycerides	0.12654	0.35432	Laboratory measurement	317
HDL Cholesterol	0.28349	0.52918	Laboratory measurement	318
C-Reactive Protein	0.16595	0.42242	Laboratory measurement	318
Leukocytes	0.25542	0.51499	Laboratory measurement	334
Hemoglobin	0.61595	0.74985	Laboratory measurement	334
Platelets	0.91006	0.94377	Laboratory measurement	334
Neutrophils	0.59209	0.74985	Laboratory measurement	317
Absolute Neutrophil Count	0.33632	0.58856	Laboratory measurement	316
Absolute Lymphocyte Count	0.64541	0.75297	Laboratory measurement	317
Absolute Monocyte Count	0.01735	0.08098	Laboratory measurement	316
HbA1c	0.51004	0.71405	Laboratory measurement	314
Progesterone	0.03861	0.13934	Laboratory measurement	316
Estradiol/E2	0.85492	0.92068	Laboratory measurement	316
Testosterone	0.79011	0.88493	Laboratory measurement	315
Androstenedione	0.00047	0.01315	Laboratory measurement	320
Dihydrotestosterone	0.42542	0.68826	Laboratory measurement	320
PSA	0.00404	0.02963	Laboratory measurement	315

Free PSA	0.03981	0.13934	Laboratory measurement	315
Free PSA %	0.00176	0.02471	Laboratory measurement	315
Vitamin A	0.08492	0.26419	Laboratory measurement	315
Vitamin E	0.95065	0.95065	Laboratory measurement	315
Zinc (Serum)	0.19364	0.45182	Laboratory measurement	313
25-Hydroxy Vitamin D	0.57205	0.74985	Laboratory measurement	315
Cortisol	0.44245	0.68826	Laboratory measurement	314
DHEA-Sulfate	0.00423	0.02963	Laboratory measurement	315
Sex Hormone Binding Globulin	0.00652	0.03651	Laboratory measurement	314
S.p. UTI (last 6 months)	0.65735	0.81603	Clinical parameter	553
S.p. urinary retention (last 6 months)	0.67203	0.81603	Clinical parameter	553
Meat consumption (days/week)	0.89596	0.89596	Clinical parameter	473
Alcohol (days/week)	0.38140	0.55165	Clinical parameter	472
Sport (hours/week)	0.37537	0.55165	Clinical parameter	463
Smoking (yes/no)	0.07088	0.20081	Clinical parameter	479
Cig. per day	0.38940	0.55165	Clinical parameter	501
Smoking (years)	0.80708	0.85752	Clinical parameter	478
Age	0.00000	0.00000	Clinical parameter	552
BMI	0.78767	0.85752	Clinical parameter	485
Positive fam. history for PC	0.31472	0.55165	Clinical parameter	490
Infertility	0.15337	0.37246	Clinical parameter	553

S.p. sterilization	0.24981	0.53086	Clinical parameter	553
Positive DRE	0.00000	0.00000	Clinical parameter	553
PSAD	0.00000	0.00000	Clinical parameter	314
PIRADS	0.00000	0.00000	Imaging parameter	543
Prostate volume	0.00031	0.00105	Clinical parameter	552

Supplementary table 2 – Median model performance per feature set

Features	Accuracy	F1	Precision	Recall
**PSA**	0.44	0.39	0.35	0.448
**+ additional lab. panel**	0.48	0.45	0.43	0.48
**+ clinical values**	0.52	0.5	0.49	0.53
**+ MRI results**	0.56	0.54	0.56	0.56
**MRI + PSA**	0.53	0.51	0.55	0.53
