# Supplementary material for: Integrative analysis of blood biomarkers and clinical variables improves early detection of aggressive prostate cancer
Source: Sci Rep. 2025 Apr 23;15:14071. doi: 10.1038/s41598-025-98980-3 (PMC12018954; doi:10.1038/s41598-025-98980-3)

Supplementary figure 1 – Associations of laboratory, clinical and imaging parameters with the presence of ISUP≥2 prostate cancer

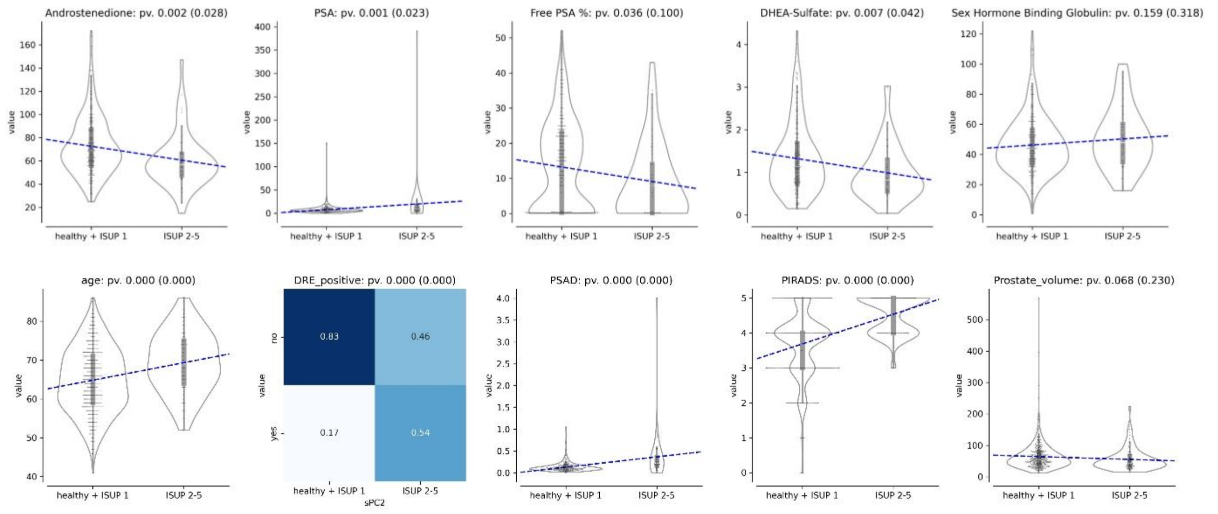

Supplement: Supplementary file 2 — Supplementary Material 2 [file 41598_2025_98980_MOESM2_ESM.pdf]
